# TurboFold: Iterative probabilistic estimation of secondary structures for multiple RNA sequences

**DOI:** 10.1186/1471-2105-12-108

**Published:** 2011-04-20

**Authors:** Arif O Harmanci, Gaurav Sharma, David H Mathews

**Affiliations:** 1 Department of Electrical and Computer Engineering, University of Rochester, Rochester, NY, USA; 2 Department of Biochemistry and Biophysics, University of Rochester Medical Center, Rochester, NY, USA; 3 Department of Biostatistics and Computational Biology, University of Rochester Medical Center, Rochester, NY, USA; 4 Center for RNA Biology, University of Rochester, Rochester, NY, USA

## Abstract

**Background:**

The prediction of secondary structure, i.e. the set of canonical base pairs between nucleotides, is a first step in developing an understanding of the function of an RNA sequence. The most accurate computational methods predict conserved structures for a set of homologous RNA sequences. These methods usually suffer from high computational complexity. In this paper, TurboFold, a novel and efficient method for secondary structure prediction for multiple RNA sequences, is presented.

**Results:**

TurboFold takes, as input, a set of homologous RNA sequences and outputs estimates of the base pairing probabilities for each sequence. The base pairing probabilities for a sequence are estimated by combining *intrinsic *information, derived from the sequence itself via the nearest neighbor thermodynamic model, with *extrinsic *information, derived from the other sequences in the input set. For a given sequence, the extrinsic information is computed by using pairwise-sequence-alignment-based probabilities for co-incidence with each of the other sequences, along with estimated base pairing probabilities, from the previous iteration, for the other sequences. The extrinsic information is introduced as free energy modifications for base pairing in a partition function computation based on the nearest neighbor thermodynamic model. This process yields updated estimates of base pairing probability. The updated base pairing probabilities in turn are used to recompute extrinsic information, resulting in the overall iterative estimation procedure that defines TurboFold.

TurboFold is benchmarked on a number of ncRNA datasets and compared against alternative secondary structure prediction methods. The iterative procedure in TurboFold is shown to improve estimates of base pairing probability with each iteration, though only small gains are obtained beyond three iterations. Secondary structures composed of base pairs with estimated probabilities higher than a significance threshold are shown to be more accurate for TurboFold than for alternative methods that estimate base pairing probabilities. TurboFold-MEA, which uses base pairing probabilities from TurboFold in a maximum expected accuracy algorithm for secondary structure prediction, has accuracy comparable to the best performing secondary structure prediction methods. The computational and memory requirements for TurboFold are modest and, in terms of sequence length and number of sequences, scale much more favorably than joint alignment and folding algorithms.

**Conclusions:**

TurboFold is an iterative probabilistic method for predicting secondary structures for multiple RNA sequences that efficiently and accurately combines the information from the comparative analysis between sequences with the thermodynamic folding model. Unlike most other multi-sequence structure prediction methods, TurboFold does not enforce strict commonality of structures and is therefore useful for predicting structures for homologous sequences that have diverged significantly. TurboFold can be downloaded as part of the RNAstructure package at http://rna.urmc.rochester.edu.

## Background

The discovery that RNA can directly regulate chemical reactions in a cell without being translated into, or coding for, a protein has radically altered the understanding of RNA function [1,2]. Many types of such non-coding RNAs (ncRNAs) have been identified, with roles in diverse cellular activities [3,4] and it is predicted that numerous ncRNAs are yet to be identified [4-8].

Correct determination of the secondary structure of a ncRNA, i.e., the canonical base pairing interactions between the nucleotides, is important for understanding the chemical basis for its function [9]. In addition, accurate prediction of RNA secondary structure also improves computational methods that scan genomes for novel ncRNA genes [4,10-14] because these methods utilize structure prediction to test for conserved secondary structure across genomes, which, in turn suggests that the sequence regions corresponding to conserved structural regions form homologous ncRNA genes.

A number of alternative techniques have been proposed for RNA secondary structure prediction - a process that is commonly referred to as RNA folding [15,16]. For folding a single RNA sequence, the state of the art method utilizes a thermodynamic model that predicts molecular stability for a given set of base pairing interactions using a nearest neighbor model [17-20]. When multiple RNA homologs that share a common secondary structure are available, significantly higher accuracy can be obtained by folding these multiple sequences together to find the conserved structure. In fact, *comparative sequence analysis *methods [21] that utilize a large number of homologs for RNA folding, currently offer the most accurate prediction of secondary structure. Comparative sequence analysis takes as input multiple homologous RNA sequences and predicts a consensus secondary structure. The analysis is an iterative process, where the sequences are aligned and conserved base pairs are identified between columns of the alignment. Then the pairing information is utilized to refine the alignment of the sequences in the next iteration. Comparative sequence analysis aims at combining the folding of individual sequences and the alignment between the sequences to determine the consensus structure. The method is, however, manual and time consuming. Computational methods for structure prediction using multiple homologous sequences can be thought of as attempts to automate comparative sequence analysis, typically with a much smaller number of input sequences. A recent comprehensive review of computational methods for structure prediction for multiple sequences can be found in [22].

**Figure 1:**
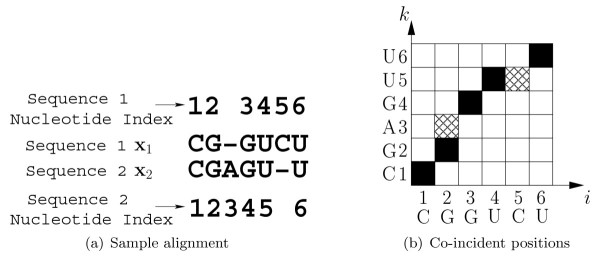
**Nucleotide coincidence**. Example illustrating nucleotide *co-incidence*. (a) a sample alignment for two hypothetical sequences **x**_1 _and **x**_2_, and (b) representation of the alignment as an array where *i *denotes the nucleotide index for sequence **x**_1 _and *k *the nucleotide index for the sequence **x**_2_. The co-incident nucleotide positions are indicated by black and cross-hatched squares, where the black squares indicate aligned positions and the cross-hatched squares indicate inserted positions.

This paper presents TurboFold, a new secondary structure prediction algorithm. TurboFold is an iterative algorithm that takes, as input, a collection of homologous RNA sequences and outputs estimates of base pairing probabilities for each of the sequences. TurboFold computes estimated base pairing probabilities for a given sequence, by using both *intrinsic information *derived from a thermodynamic nearest neighbor model for folding of the sequence and *extrinsic information *for folding of the sequence inferred from the other sequences in the input set. The extrinsic information contribution of each of the other sequences is obtained by mapping the estimated base pairing probabilities for the sequences, from the previous iteration, using posterior probabilities of nucleotide co-incidence between sequences. Two nucleotide positions (one from each of the two sequences) are *co-incident *if they are either aligned, or if one nucleotide position (from one of the sequences) occurs in an insertion in that sequence that begins at a nucleotide position aligned with the second nucleotide position (from the other sequence) [23]. Co-incidence is illustrated in Figure 1 and a formal definition can be found in [23]. The pairwise nucleotide co-incidence probabilities are obtained by using a Hidden Markov Model (HMM) for the alignment between the sequences.

The estimated posterior probabilities of base pairing output by TurboFold can be utilized for predicting the secondary structure of the sequences, either by thresholding the probabilities to obtain structures composed of base pairs with estimated pairing probabilities higher than a desired threshold or by using the estimated posterior probabilities in a maximum expected accuracy (MEA) secondary structure prediction algorithm [24-26]. The latter algorithm is termed TurboFold-MEA. While TurboFold predicts the secondary structures for the multiple sequences collectively using information from all sequences, it does not do so with a rigid definition of common secondary structure for the collection of sequences. Thus TurboFold permits variable folding domains that are seen in some of the sequences and not in others, a scenario that is not uncommon in ncRNA sequences that are homologous despite the minor variations in their secondary structure topology.

Benchmarking results demonstrate that the base pairing probability estimates of TurboFold are more accurate than alternative methods that provide such estimates, i.e. for a given sensitivity, the base pair predictions obtained by thresholding the estimated probabilities from TurboFold have a higher positive predictive value (PPV) than the alternative methods. Secondary structure prediction using TurboFold-MEA also provides among the highest accuracy across the secondary structure prediction methods benchmarked. Specifically, for ncRNA families with significant structural variation, TurboFold-MEA has a higher sensitivity than other methods at similar PPV. For other ncRNA families, the results of TurboFold-MEA are comparable to the best performing methods. The computation time and memory requirements of TurboFold are modest and comparable to, or lower than, those for other methods with comparable accuracy, with the exception of RAF [27], which is faster.

In the next section, TurboFold is presented as an iterative algorithm that alternates between computations of a) extrinsic information and b) a modified partition function that yields estimates of posterior base pairing probabilities. Within the section, a description is also provided for methods for prediction of secondary structures from base pairing probability estimates, either by composing structures made from base pairs with estimated probabilities higher than a chosen threshold or by using the MEA methodology. The Results section benchmarks the performance of TurboFold and TurboFold-MEA against other secondary structure prediction methods with regard to structure prediction accuracy and resource (time and memory) requirements. The Discussion section presents the motivation for the proposed method and the nomenclature by exploring connections with Turbo-decoding [28] and presents an example that illustrates TurboFold's ability to allow variable structural elements across input sequences. The relation of TurboFold to existing multi-sequence methods for prediction of RNA secondary structure is also addressed within the Discussion section.

## Methods

TurboFold takes as input *K *RNA sequences denoted by **x**_1_, **x**_2_, ..., **x***_K _*or  where  denotes the set of sequence indices. The length of the *m*^th ^sequence **x***_m _*is denoted by *N_m_*. Thus the sequence **x***_m _*consists of an sequence of *N_m _*nucleotides ordered from the 5' to the 3' end, where each nucleotide takes values from the alphabet set {*A, U, G, C*} based on its identifying nitrogenous base. A secondary structure **S***_m _*on an RNA sequence **x***_m _*is represented as the set {(*i_l_, j_l_*)}*_l _*of pairs (*i_l_, j_l_*) of nucleotide indices *i_l_*, *j_l _*corresponding to the base pairs in the secondary structure, where the subscript *l *indexes the base pairs in the structure. By convention, 1 ≤ *i *<*j *≤ *N_m _*and each nucleotide position can participate in at most one base pair. Furthermore, as is common, for computational reasons, it is assumed that the base pairs within a structure satisfy the pseudoknot free condition, i.e. for any four nucleotide indices 1 ≤ *i*_1 _<*i*_2 _<*j*_1 _<*j*_2 _≤ *N_m_*, both (*i*_1_, *j*_1_) and (*i*_2_, *j*_2_) cannot be base pairs in **S***_m_*.

The steps in TurboFold are listed in Algorithm 1. The ensuing description first provides a high-level overview which is followed by details of the individual modules within the algorithm. The notational convention denotes probabilities by *π *and matrices of probability entries by **Π**. Terms analogous to, but not strictly, probabilities are denoted as  and , respectively, in their scalar and matrix forms. The association of these terms with a sequence or a pair of sequences is indicated by adding superscripts comprised of a single sequence index or a two-tuple of sequence indices. Pre-subscripts of *p *and *c *indicate that they are associated with pairing and co-incidence events, respectively. Finally, if required, a pre-superscript denotes the iteration index.

Prior to commencing the iterations, pairwise posterior *co-incidence *probability matrices *_c_***Π**^(*s*,*m*) ^and pairwise sequence identities *ψ*_*m*,*s *_are computed for each pair of sequences (*m*, *s*), *m*, , *m *≠ *s*. Specifically, *_c_***Π**^(*m,s*) ^is an *N_m _*× *N_s _*matrix whose *ik*^th ^entry *_c_π*^(*m*,*s*) ^(*i*, *k*) is the posterior probability that nucleotide at index *i *in **x***_m _*is co-incident with the nucleotide at index *k *in **x***_s_*. The sequence identity, *ψ*_*m*,*s*_, is computed as the fraction of positions, along the maximum likelihood alignment path, in which the nucleotides for sequence **x***_m _*and **x***_s _*match. The posterior co-incidence probability matrices *_c_***Π**^(*s,m*) ^and sequence identity *ψ*_*m*,*s *_are computed efficiently in TurboFold using a pairwise alignment Hidden Markov Model (HMM) [23,29], which requires *O*(*N_m_N_s_*) operations and storage for each sequence pair.

**Figure 2:**
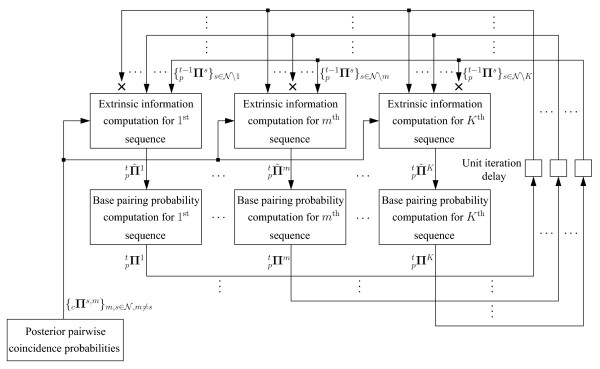
**Flowchart for iterative computation of probabilities of base pairing and extrinsic information**. The pairwise coincidence probabilities are computed once during initialization. Iteration *t *starts with computation of the extrinsic information for the sequences, utilizing the base pairing probabilities  computed in the previous iteration. Note that the extrinsic information computation for a sequence at iteration *t *does not utilize its own base pairing probabilities computed at iteration (*t *- 1). This is shown in the figure by an '×' symbol between the arrow that represents the base pairing probability of the sequence and the extrinsic information computation block for the sequence. The first iteration starts with initialization of extrinsic information of each sequence to 1. (*η *+ 1) total iterations are performed.

Once similarities and posterior co-incidence probabilities are available, TurboFold proceeds with iterations indicated in Algorithm 1, where *t *denotes the iteration count, commencing at *t *= 0. The *t*^th ^iteration computes base pairing probability matrices  for each sequence  using, as input, the base pairing probability matrices  computed in the previous iteration. Specifically,  is an *N_m _*× *N_m _*lower triangular matrix, whose *ij*^th ^element  represents the *t*^th ^iteration estimate of the probability that in the secondary structure of **x***_m _*the nucleotides at indices *i *and *j *in the sequence are base-paired. The computation of the base pairing probability matrix  comprises two steps, details of which follow the overview of the algorithm flow. The first step computes a lower triangular *N_m _*× *N_m _*extrinsic information matrix , using base pairing probability matrices  for all sequences other than **x***_m _*from the previous iteration. The notation  denotes the set of indices obtained by deleting *m *from the full set of indices . The second step computes a modified partition function that combines the extrinsic information with the nearest neighbor thermodynamic model to obtain the base pairing probability matrix . The algorithm terminates after (*η *+ 1) iterations where the first iteration (*t *= 0) corresponds to an initialization step where base pairing probabilities  are computed with the extrinsic information set to unity for all sequences. The overall process is illustrated in Figure 2 in a flow chart format that highlights the iterative nature of the algorithm and the analogy with Turbo-decoding in digital communications.

### Extrinsic Information Computation

**Figure 3:**
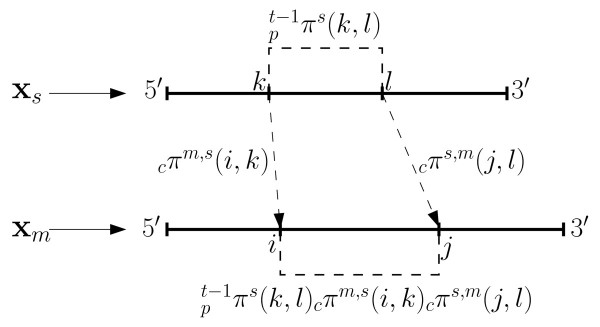
**Illustration of induced base pairing proclivities formulated in (1)**. Heavy lines represent the sequences with nucleotide positions indicated by thick dashes. The dashed lines between *k *and *l*, and between *i *and *j *represent potential base pairing interactions. The probability of base pairing between *k *and *l *is shown as *_p_π^s^*(*k*, *l*). The dashed arrows indicate the co-incidence of *k *with *i *and *l *with *j*. The base pairing probability of nucleotides at *k *and *l *in **x***_s _*induces base pairing proclivities for nucleotides at *i *and *j *in **x***_m _*based on alignment co-incidence probabilities *_c_π*^(*m*,*s*)^(*i*, *k*) and *_c_π*^(*m*,*s*)^(*j*, *l*) resulting in an induced proclivity .

The process for computing extrinsic information  for the *m*^th ^sequence **x***_m _*in the *t*^th ^iteration is outlined next. First values for base pairing *proclivity *for the sequence **x***_m _*induced by each of the other sequences are computed. Specifically, for each , an *N_m _*× *N_m _*lower triangular matrix  is evaluated. The *ij*^th ^entry of  is computed as in (1) and characterizes the proclivity for base pairing between the nucleotides at indices *i *and *j *in the sequence **x***_m_*, as induced by: a) the base pairing probability matrix  for the sequence **x***_s _*in the (*t *- 1)*^th ^*iteration and b) the alignment posterior co-incidence probability matrix *_c_***Π**^(*m*,*s*)^. Equation (1) can be intuitively understood by referring to Figure 3. Multiplying , which represents the most recent estimate of the probability that nucleotides at indices *k *and *l *are paired in **x***_s_*, with the probabilities *_c_π*^(*m*,*s*)^(*i*, *k*) and *_c_π*^(*m*,*s*)^(*j*, *l*) that the nucleotide indices *k *and *l *in **x***_s _*are co-incident with the nucleotide indices *i *and *j*, respectively, in **x***_m _*yields an estimate for the proclivity of base pairing induced from the index 2-tuple (*k*, *l*) in **x***_s _*onto the index 2-tuple (*i*, *j*) in **x***_m_*. This estimate is exactly the term listed in the summation on the right hand side of (1). The summation itself represents an aggregation of the proclivity estimates across the different possible base pairs (*k*, *l*) in **x***_s_*.

In (1), the induced proclivity terms for which either of the two co-incidence probabilities are small can be excluded from the summation in order to reduce the computational load, without incurring a significant(1)

performance penalty. This is indicated in (1) by constraining the indices *k *and *l *to constraint sets  and , respectively, where  denotes the set of indices for which the posterior co-incidence probabilities *_c_π *^(*m*,*s*)^(*i*, *k*) exceed a chosen, sufficiently low, significance threshold, and  is similarly defined. The computation of these sets of constrained co-incident indices is described in detail in [23]. If, over all choices of sequence pairs (*m*, *s*), the average number of elements in the set  (and ) is *d*, then the computation of a term in one of the matrices  requires (*d*^2^) operations on average. It is worth noting that without the constraints for indices *k *and *l*, the evaluation of induced probabilities in (1) could be expressed as two matrix multiplications, , which would require  operations per entry.

The use of co-incidence, rather than alignment, probabilities for the generation of extrinsic information is motivated by the fact that the coincidence probabilities, which are the sum of probabilities for matching, insertion and deletion events in the alignment, propagate pairing proclivities to inserted base pairs that change the lengths of helices, whereas alignment probabilities would restrict the extrinsic information to only the conserved base pairs.

**Figure 4:**
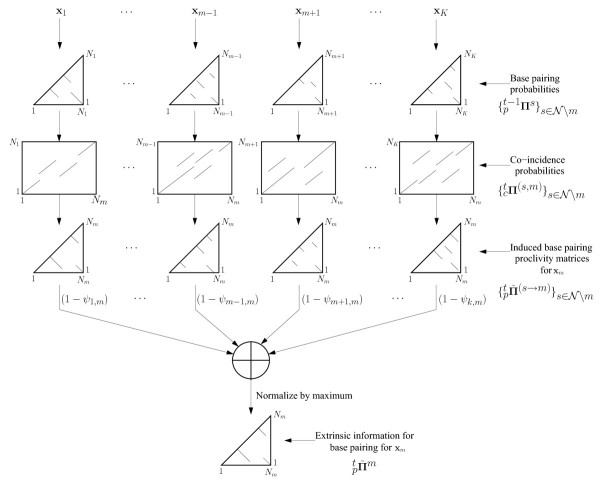
**Flowchart for the computation of extrinsic base pairing information for x*_m_***. The induced base pairing proclivity matrices, denoted by , are computed utilizing the base pairing probability matrices,  (lower triangular matrices), and the posterior co-incidence probabilities {_*c*_**Π**^(*m*,*s*)^}. The extrinsic information, , is computed as the normalized weighted sum of the induced proclivity matrices. The lines in pairing probability matrices represent helices composed of pairs with relatively high pairing probability. Lines in co-incidence probability matrices represent relatively probable regions of sequence alignment.

Utilizing the induced base pairing proclivity matrices, the extrinsic information for base pairing for **x***_m _*is computed as:(2)

where  is a normalizing factor chosen to ensure that the maximum value in  is unity. The factor (1 - *ψ*_m,s_) in (2) weights the contribution of **x***_s _*to the extrinsic information for **x***_m _*using the sequence identity, *ψ*_*m,s*_, for sequences **x***_s _*and **x***_m_*. The sequences that are highly similar to **x***_m_*, have a lower contribution to extrinsic information than those with lower similarities. In the extreme case that a sequence **x***_s _*is the same as the sequence **x***_m_*, *ψ*_*m*,*s *_= 1, and the weighting factor (1 - *ψ*_*m*,*s*_) sets the contribution of the extrinsic information to zero, which is desirable because in this setting, sequence **x***_s _*contributes no useful extrinsic information for folding of **x***_m_*. Figure 4 illustrates the process for computing extrinsic information  for the *m*^th ^sequence **x***_m _*in the *t*^th ^iteration in a flow chart format.

The aggregation of proclivity matrices and normalization of the aggregate proclivity matrix for computation of extrinsic information for **x***_m _*requires  and  operations, respectively. The total number of computations for evaluating the extrinsic information for all sequences, utilizing (2) for each sequence, is:(3)

The asymptotic time complexity of extrinsic information computation for all sequences is *O*(*K*^2^*d*^2^*N*^2^), where *N *is the longest sequence length. The memory complexity is *O*(*KN*^2^) for storage of the extrinsic information matrix for the set of *K *sequences.

### Modified Partition Function for Updating Base Pairing Probabilities

At the *t*^th ^iteration, an updated estimate of the base pairing probability matrix  for the sequence **x***_m _*is obtained from the extrinsic information  and the nearest neighbor thermodynamic model for **x***_m_*, which, in TurboFold, encapsulates the intrinsic information for folding of **x***_m_*. A modified Boltzmann distribution is used to model the probability distribution of secondary structures on **x***_m_*, where the probability of structure **S***_m _*is modeled as(4)

where *R *is the gas constant, *T *is the absolute temperature, and(5)

is a modified free energy change for structure **S***_m _*(on **x***_m_*). Here Δ*G*^0^(**S***_m_*) is the Gibbs free energy change of folding for **S***_m_*, which is obtained using the nearest neighbor thermodynamic model with the free energy parameters from [17,19]. The extrinsic information for base pairing contributes to the modified free energy in (5) as a pseudo-free energy term for each base pair in **S **and *γ *denotes the relative contribution of this extrinsic information relative to the intrinsic information represented by Δ*G*(**S***_m_*). The denominator in (4) represents a modified partition function for **x***_m _*defined as:(6)

The probability of base pairing between nucleotides at indices *i *and *j *in **x***_m _*is formulated as the summation of the probabilities of structures of **x***_m _*that contain (*i*, *j*):(7)

The base pairing probability matrix  is computed efficiently via a modification of the dynamic programming algorithm for partition function calculation [30,31] that uses the nearest neighbor thermodynamic model. Specifically, the pseudo-free energy term in (5) represents an *a priori *probability  for the base pair (*i*, *j*), which in the modified dynamic programming algorithm contributes an addition of the pseudo free energy  when considering pairing between nucleotides (*i*, *j*). The computation of modified partition function for all sequences has *O*(*KN*^3^) time complexity and *O*(*KN*^2^) memory complexity, where *N *is the longest sequence length.

### Structure Prediction Utilizing the Base Pairing Probabilities

The base pairing probabilities computed by TurboFold, , are utilized for structure prediction via two methods. The first method thresholds the base pairing probability matrix to determine the base pairs whose estimated probabilities are higher than a significance level *P*_thresh_. This yields a corresponding structure(8)

composed of base pairs deemed significant. Any choice of *P*_thresh _greater than 0.5 guarantees that  is a valid secondary structure [31]. For *P*_thresh _≤ 0.5,  may contain base pairs that form pseudoknots or may contain multiple base pairs for a nucleotide.

The second method, TurboFold-MEA, predicts the structures via maximum expected accuracy algorithm [24-26]. Given the base pairing probabilities  for **x***_m_*, the maximum expected accuracy structure is determined as in (10), where  is the probability that nucleotide at *i *is not paired with any other nucleotides.  is computed as:(9)

The computation of maximum expected accuracy structure is accomplished via a dynamic programming algorithm. The prediction of structures for all the sequences has *O*(*KN*^3^) time and *O*(*KN*^2^) memory complexity, where *N *is the length of longest sequence.(10)

### Time and Space Complexity

The time and space complexity of TurboFold can be described in terms of the operations required for the one time initialization and the operations required for the *η *computationally identical iterations. For the initialization, the estimation of posterior co-incidence probability matrices and the pairwise sequence identities for all sequence pairs requires *O*(*K*^2^*N*^2^) computations. In order to store the co-incidence probability matrices computed in the initialization, *O*(*K*^2^*dN*) memory is required. Over the *η *iterations, for all the sequences, updates of the extrinsic information require *O*(*ηK*^2^*N*^2^*d*^2^) computations and the modified partition function evaluations require *O*(*ηKN*^3^) computations. The storage requirement for the iterations is *O*(*KN*^2^). These requirements for TurboFold can be contrasted with Sankoff's algorithm, which requires *O*(*N*^3^*d^K^*) computations and *O*(*N*^2^*d^K^*) memory, when used with a banded constraint on the nucleotide alignments for reducing computation by "cutting corners" [32]. Thus, the time and memory requirements for Sankoff's algorithm increase exponentially with increasing number of input sequences, whereas the time requirement for TurboFold increases proportional to the square of the number of input sequences, and memory requirement increases linearly with the number of input sequences.

It should be noted that, in each iteration, the base pairing probability computations for each sequence are performed independently. Therefore the base pairing probabilities for all sequences can be computed in parallel using *K *processors. In the current implementation of TurboFold, the user can specify the number of threads that will be used to compute the base pairing probabilities in parallel. The POSIX threads library is utilized for implementation of parallel computations.

### Measures for Accuracy of Predicted Structures

The structure prediction accuracy is evaluated in terms of sensitivity and positive predictive value (PPV) of the predictions. For a sequence **x***_m_*, the sensitivity of the predicted structure is the ratio of number of correctly predicted base pairs to the number of base pairs in the known structure and the PPV is the ratio of the number of correctly predicted base pairs to the number of base pairs in the predicted structure. A base pair between nucleotides at *i_m _*and *j_m _*in the predicted structure is assumed to be correctly predicted if there is a base pair (*i_m_*, *j_m_*) or (*i_m _*- 1, *j_m_*) or (*i_m _*+ 1, *j_m_*) or (*i_m_*, *j_m _*-1) or (*i_m_*, *j_m _*+ 1) in the known structure, which is consistent with prior methodology for accuracy assessment [18,33,34]. This scoring reflects the uncertainty in structure determination by comparative analysis and thermal fluctuations in structure.

### Selection of Parameters

**Figure 5:**
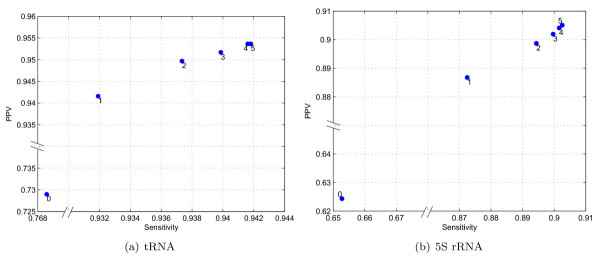
**Sensitivity versus PPV for TurboFold as a function of iteration count**. Plots of sensitivity versus PPV for structure prediction by TurboFold with increasing number of iterations, *η*, for: (a) tRNA and (b) 5S rRNA training datasets with *K *= 5. Note the discontinuities in the axes which are indicated by the breaks. The 0^th ^iteration utilizes no extrinsic information and is therefore the average accuracy of single-sequence MEA structure prediction.

The number of iterations, *η*, and relative weight of extrinsic information, *γ*, in the modified free energy in (5) are selected empirically based on experiments. To select the parameters, the prediction accuracy of TurboFold is evaluated with different values for *γ *and *η *on four training datasets. The datasets include two tRNA datasets from the compilation of tRNA sequences and structures by Sprinzl [35] and two 5S rRNA datasets from the 5S Ribosomal RNA Database [36], respectively. For each family, 250 sequences are chosen randomly and divided into combinations of *K *sequences, where the process is repeated independently for *K *= 5 and *K *= 10, yielding two training datasets per family (corresponding to *K *= 5 and *K *= 10). The number of iterations, *η*, is varied from 0 to 5. Figure 5 shows the plots of sensitivity versus PPV of structure prediction via TurboFold-MEA with varying *η *for tRNA and 5S rRNA datasets. The reported sensitivity and PPV for each family is the average sensitivity and PPV of predictions over *K *= 5 dataset. The average sensitivity versus PPV plots for changing *η *over the *K *= 10 dataset are included in the Supplementary Data (Additional file 1). Increasing the number of iterations increases both sensitivity and PPV for both families. Increasing the number of iterations, however, also linearly increases the computation time required for TurboFold. Because the increases in sensitivity and PPV are marginal for *η *> 3, the number of iterations is chosen as *η *= 3.

**Figure 6:**
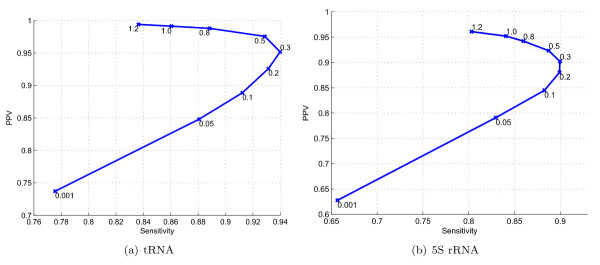
**Sensitivity versus PPV for TurboFold as a function of *γ*/*RT***. Plots of sensitivity versus PPV for structure prediction by TurboFold with increasing value of *γ*/*RT *for: (a) tRNA and (b) 5S rRNA training datasets with *K *= 5.

For selecting *γ*, the structure prediction accuracy of TurboFold-MEA is evaluated, utilizing *η *= 3, with a set of values for *γ *such that *γ*/*RT *∈ {0.001, 0.05, 0.1, 0.2, 0.3, 0.5, 0.8, 1.0, 1.2}. Figure 6 shows the plots of sensitivity versus PPV of structure prediction with changing value of *γ*/*RT *over *K *= 5 datasets. The sensitivity versus PPV plot for predictions over *K *= 10 datasets are included in Supplementary Material (Additional file 1). Increasing *γ *increases PPV for both datasets. Furthermore, *γ*/*RT *≈ 0.3 maximizes sensitivity of structure prediction for both datasets. Increasing *γ *above 0.3 *RT *introduces a tradeoff between sensitivity and PPV. *γ *= 0.3 *RT *is therefore used in the TurboFold benchmarks.A version of the TurboFold source code that was used to obtain the benchmarking results presented here can be found as Additional File 2

## Results

Three sets of experiments are performed for comparing TurboFold with other programs: 1) Experiments for assessing accuracy of structures predicted from thresholding of base pairing probabilities as computed by TurboFold; 2) Experiments for assessing accuracy of structures predicted from TurboFold-MEA; 3) Experiments for comparing time and memory requirements of TurboFold with other programs. Datasets for benchmarking experiments are generated as follows: 200 RNase P sequences are randomly selected from the RNase P Database [37], then the sequences are split into sets of *K *sequences such that 2 ≤ *K *≤ 10. The average sequence length is 336 nucleotides and the average pairwise identity, as determined from the alignments computed by ClustalW 2.0.11 [38], is 50%. The random selection and division into combinations of *K *sequences (for 2 ≤ *K *≤ 10) is also performed with 200 tmRNA sequences [39,40] (average length of 366 nucleotides and average pairwise identity of 45%), and 30 telomerase RNA sequences [41] (445 nucleotides and 54% pairwise identity), 400 SRP sequences from the SRPDB [42] (187 nucleotides and 42% pairwise identity), 400 tRNA sequences from the compilation of tRNA sequences by Sprinzl et al. [35] (77 nucleotides and 47% pairwise identity), and 400 5S rRNA sequences from the 5S Ribosomal RNA database [36] (119 nucleotides and 63% pairwise identity). This procedure yields 9 datasets for each family and 54 datasets in total. The datasets are available as Additional File 3

### Performance Benchmarks for Estimated Base Pairing Probabilities

The accuracy of structures predicted by thresholding of base pairing probabilities estimated by TurboFold, is compared with three other methods that estimate base pairing probabilities:

1. LocARNA [43] is structural alignment algorithm for multiple sequences that utilizes pairwise structural alignment computations progressively for prediction of the structural alignment. Version 1.5.2a is utilized, with Vienna RNA Software Package version 1.8.4, in probabilistic mode to generate base matching probabilities with consistency transformation ('-probabilistic -consistency-transformation' option). Given *K *input sequences, the single sequence reliabilities as computed by LocARNA are utilized as estimates of base pairing probabilities.

2. RNAalifold [44] is a structure prediction algorithm that takes a sequence alignment of the input sequences. The structures are predicted via maximization of a score that is based on free energy changes and covariation from the sequence alignment. RNAalifold also estimates the base pairing probabilities for sequences via computation of a partition function for the alignment. The version included in Vienna RNA Software Package version 1.8.4 is utilized with command line option '-p' for computation of base pairing probabilities with ClustalW 2.0.11 [38] for computation of input sequence alignment.

3. Single sequence partition function computation [30,31], which computes the base pairing probabilities of a given RNA sequence in the equilibrium ensemble of secondary structures. The partition function computation as implemented in RNAstructure version 4.5 [31,45] are utilized in benchmark experiments.

**Figure 7:**
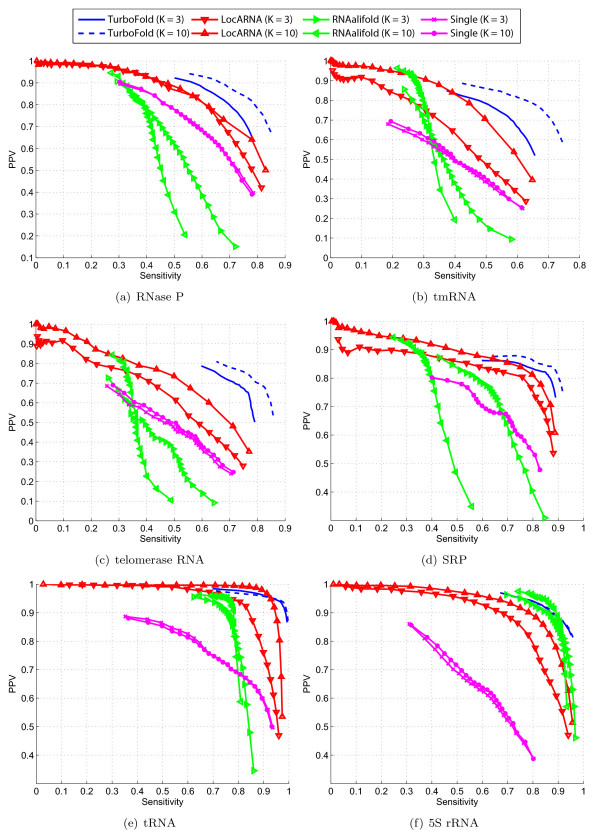
**Sensitivity vs PPV of TurboFold compared against LocARNA, RNAalifold, and single sequence partition function**. Sensitivity versus PPV of base pairs with probabilities, as computed by single partition function, LocARNA, RNAalifold and TurboFold, over: (a) RNase P, (b) tmRNA, (c) telomerase RNA, (d) SRP, (e) tRNA, and (f) 5S rRNA. The RNase P, tmRNA, telomerase RNA, SRP, tRNA, and 5S rRNA datasets have sequence similarity of 50%, 45%, 54%, 42%, 47%, and 63%, respectively.

For each method, for a given threshold *P*_thresh_, the structure formed by base pairs whose estimated probabilities exceed *P*_thresh_, is computed. The sensitivity and PPV of this structure are then evaluated with respect to the known structure. The threshold probability *P*_thresh _is varied from 0.04 to 0.96 in steps of 0.04 to obtain number of sensitivity vs PPV points which are then plotted along a curve, one for each method. Figure 7 shows the plots of sensitivity versus PPV for the four methods over the datasets for two choices of number of sequences, *K *= 3 and *K *= 10.

For the RNase P, tmRNA, telomerase RNA, and SRP datasets, TurboFold has higher sensitivity for a fixed PPV, and higher PPV for a fixed sensitivity than the other methods. In addition, the PPV versus sensitivity plot for TurboFold approaches the top right corner, corresponding to ideal (sensitivity, PPV) = (1.0, 1.0), closer than the other three methods evaluated. The accuracy of TurboFold and LocARNA are comparable over tRNA datasets. Over 5S rRNA datasets, the accuracy of TurboFold is comparable to that of RNAalifold. The prediction accuracy of RNAalifold, however, depends significantly on the accuracy of the input alignment computed by ClustalW. Over datasets with high average pairwise identity, which are easier to align, predictions of RNAalifold are higher in accuracy than over datasets with lower average pairwise identity. Figure 7 illustrates this: Compared to other methods, the accuracy of RNAalifold predictions is highest for the 5S rRNA, whose average pairwise identity is significantly higher than average identities of other datasets. Additionally, the accuracy of RNAalifold for the *K *= 10 dataset is lower than for the *K *= 3 datasets when average sequence identity is low. TurboFold demonstrates a better performance with *K *= 10 than with *K *= 3 for all sequence families, as expected.

### Structure Prediction Accuracy of TurboFold-MEA

The structure prediction accuracy of TurboFold-MEA is compared with eight other structure prediction methods listed below.

1. RAF [27] is a structural alignment algorithm that utilizes progressive pairwise alignments to predict the structural alignment. RAF utilizes a simple scoring scheme based on base pairing probabilities (as computed by CONTRAfold 2.02 [25]), alignment probabilities (as computed by CONTRAlign 2.01 [46]), and a set of weights learned from a dataset of multiple structural alignment dataset for structural alignment prediction. Version 1.0 is utilized with the default command line option for prediction ('-predict' option).

2. LocARNA [43] Version 1.5.2a (with Vienna RNA Software Package version 1.8.4) is utilized.

3. CentroidAlifold [47,48] is a structural alignment method that takes an input sequence alignment and combines the base pairing information and input sequence alignment to predict structures for each sequence. The input sequence alignment is generated by ClustalW 2.0.11 [38].

4. RNASampler [49] is an iterative sampling algorithm that predicts conserved helices in input sequences for structure prediction. RNA Sampler was used with default options.

5. RNAcast [50] analyzes the folding space of input sequences in terms of abstract shapes and finds the optimal abstract shape that is common for all the structures and uses the optimal shape to generate consensus secondary structure. RNAcast is used with 40% free energy energy cut-off threshold, as in [34], because RNAcast fails to determine consensus structures for some datasets for higher thresholds.

6. FOLDALIGNM [51] is a method for progressive structural alignment of RNA sequences. FOLDALIGNM version 1.0.1 is run with FOLDALIGN version 2.1.1 [52]. The java heap space is set to 10 gigabytes (with '-x 10000' option).

7. MASTR [53] is a Markov chain Monte Carlo algorithm for structural alignment of a given set of RNA sequences. The default command line options are used for MASTR.

8. MXScarna [54] is a method for structural alignment of multiple RNA sequences. MXScarna progressively aligns the sequences using an efficient pairwise structural alignment algorithm for determining the set of stems in the sequences that optimizes a scoring function evaluated from precomputed probabilities of base pairing and alignment. Version 2.1 is used in the predictions.

9. CentroidHomfold [55] is a method that takes as input a target RNA sequence and (*K *- 1) sequences that are homologous to the target sequence and predicts a structure for the target sequence. For an input set of *K *sequences, predictions for each sequence are obtained by running CentroidHomfold *K *times with each of the sequences serving as the target sequence once with the remaining (*K *- 1) sequences as the homologous sequences. CentroidHomfold version 1.0 is used.

10. Free energy minimization [19,45] as implemented in RNAstructure version 4.5 is used for single sequence structure predictions.

Structure prediction accuracies of all the methods are evaluated over the 54 testing datasets. Some of the methods failed to complete on some of the datasets because of rather large memory requirements. These methods are therefore excluded from the reported results for the corresponding cases in the following description.

**Figure 8:**
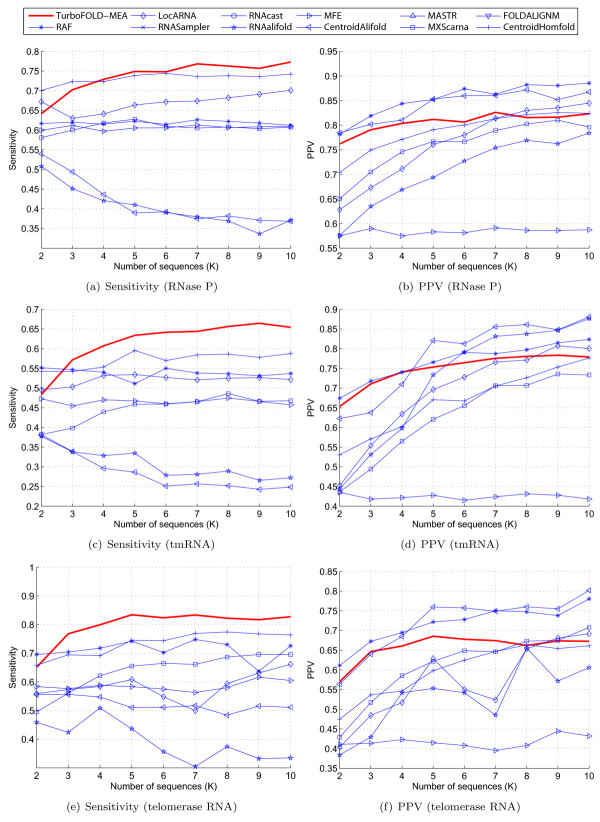
**Sensitivity and PPV of structure prediction vs number of sequences**. Part I. Sensitivity and PPV of structure prediction versus number of sequences for RNase P ((a) and (b), respectively), tmRNA ((c) and (d), respectively), and telomerase RNA ((e) and (f), respectively) datasets. Methods that did not complete execution for a dataset because memory requirements exceeded available resources are excluded from the corresponding plots.

Figure 8 shows the sensitivity versus number of sequences (K) and PPV versus number of sequences for the RNase P, tmRNA and telomerase RNA testing datasets. Among the methods benchmarked, TurboFold-MEA performs the best in terms of sensitivity for all these datasets except for RNase P dataset, where TurboFold-MEA and CentroidHomfold perform comparably in sensitivity. In addition, TurboFold-MEA is one of the best four methods in terms of PPV for all the RNase P and the telomerase RNA testing datasets.

**Figure 9:**
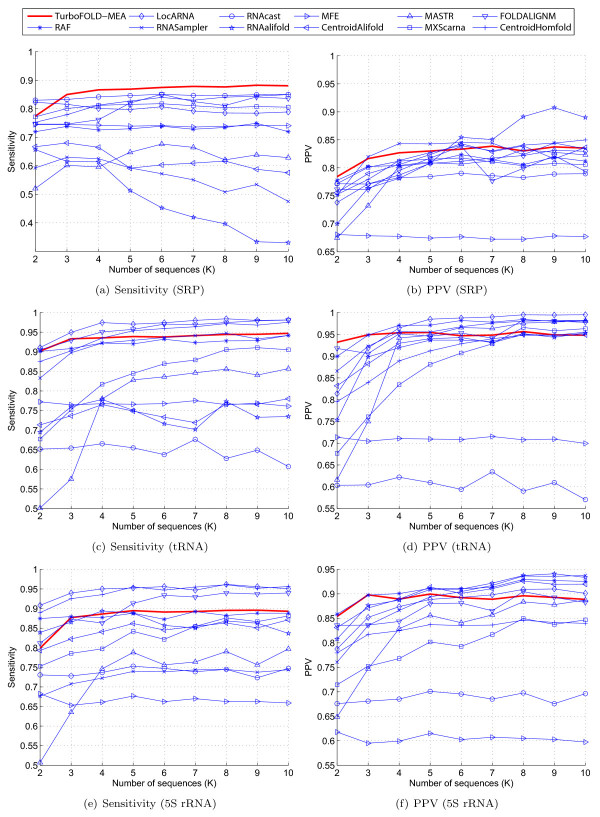
**Sensitivity and PPV of structure prediction vs number of sequences**. Part II. Sensitivity and PPV of structure prediction versus number of sequences for SRP ((a) and (b), respectively), tRNA ((b) and (c), respectively), and 5S rRNA ((d) and (e), respectively) datasets. Methods that did not complete execution for a dataset because memory requirements exceeded available resources are excluded from the corresponding plots.

Figure 9 shows the sensitivity versus number of sequences and PPV versus number of sequences for the SRP, tRNA, and 5S rRNA datasets. For the SRP datasets, TurboFold-MEA performs the best in terms of sensitivity and performs comparable to the other methods in terms of PPV. Sensitivity and PPV of TurboFold-MEA predictions for the tRNA and the 5S rRNA datasets are comparable to the other methods. The relative sensitivity and PPV of TurboFold-MEA with respect to other methods does not change compared to the plots in Figures 8 and 9, when the results are separated into groups based on average sequence identity though all methods have higher sensitivity for datasets with higher sequence identity compared to datasets with lower identity. Results are included in Supplementary data.

### Comparison of Time and Memory Requirements

The methods are also compared in terms of memory and time requirements. For this purpose, three datasets are generated by randomly selecting 50 RNase P sequences and then dividing the RNase P sequences into *K *= 3, *K *= 5, and *K *= 10 sequence combinations. It should be noted that the range of the run times required by all the methods is large (from several seconds to many hours). The timing and memory benchmarks are performed over the datasets chosen from RNase P family because for these datasets, the time and memory requirements for all methods are large enough to enable reliable estimation. The experiments are performed on a compute cluster for which each node is equipped with two quad-core Intel Xeon 3.0 GHz processors and 16 GB of main memory running Red Hat Enterprise Linux Server release 5.4. Table 1 shows the time requirements of the methods that executed successfully. The memory requirements of FoldAlignM, MASTR, RNAcast and RNA Sampler exceeded the available main memory on the utilized node. For each method, the reported time requirement is the CPU time spent by the method, as reported by the portable batch system (PBS) running on the cluster. Comparing the two multi-sequence methods that provide base pairing probability estimates and do not require an input alignment, it can be seen that TurboFold is faster than LocARNA. RNAalifold has the smallest runtime on all the datasets. TurboFold-MEA runs slower than all methods except LocARNA. In addition, the computational requirements of LocARNA scale up fastest as the number of sequences *K *increases. RAF also shows a similar behavior, but the scaling is not as steep as LocARNA. Increasing the number of input sequences increases the time requirements of TurboFold-MEA though these requirements increase by a smaller scaling factor compared to the increase for RAF and LocARNA.

**Table 1:** Computation time

	Runtime (seconds) for		
	***K *= 3**	***K *= 5**	***K *= 10**

TurboFold-MEA	136.75	277.9	517.0

RAF	8.25	50.8	214.6

LocARNA	746.44	2815.9	11395.8

CentroidAlifold	2.0	3.7	6.8

RNAalifold	0.2	0.3	0.6

MXScarna	1.5	2.9	5.8

CentroidHomfold	15.9	54.2	210.0

The time requirements (in seconds) of methods on timing/memory datasets. Each column shows the time requirements of the methods on a dataset, indicated by number of sequences.

Table 2 shows the memory usage of each method. For each experiment, the memory usage is determined from the memory reported by the PBS. TurboFold has lower memory requirements than LocARNA and RAF. CentroidAlifold and RNAalifold have the lowest memory requirements. The memory requirements of all the methods increase with increasing number of sequences. As in Table 1, as the number of input sequences increases, memory usage increases by the largest scaling factor for RAF and LocARNA.

**Table 2:** Memory usage

	Memory Usage (Megabytes) for		
	***K *= 3**	***K *= 5**	***K *= 10**

TurboFold-MEA	111.4	161.9	235.1

RAF	184.1	381.1	518.2

LocARNA	204.2	195.9	296.3

CentroidAlifold	48.4	49.6	50.1

RNAalifold	49.5	49.1	49.7

MXScarna	47.0	46.9	47.1

CentroidHomfold	52.6	55.6	51.2

The memory usage of the methods on timing/memory datasets. Each column shows the memory usage of the methods on one dataset, indicated by number of sequences.

## Discussion

The computation of extrinsic information in TurboFold is similar to several previous approaches for combining homology information for multi-sequence alignment and structure prediction. For example, the method proposed in [55] approximates base pairing probabilities via a computation similar to the extrinsic information computation. TurboFold, however, is fundamentally different. Whereas the method in [55] is non-iterative and directly utilizes the approximated probabilities for structure prediction via a Nussinov style [56] dynamic programming algorithm, TurboFold iteratively updates the extrinsic information and recomputes probabilities of base pairing, alternating between these steps in order to refine the estimates of posterior base pairing probabilities. As shown in the Results Section, the iterative procedure offers a significant improvement over a single computation. Also, the consistency transformation [57] is utilized by LocARNA for re-estimating the alignment probabilities in the progressive alignment via a procedure similar to extrinsic information computation. This procedure, unlike the method in [55], updates only the probabilities of alignment and the structure predictions are not explicitly updated. LocARNA can, however, perform iterative refinement to update the predictions of structures and alignment. Another difference is that the other methods use posterior alignment probabilities whereas TurboFold uses posterior *co-incidence *probabilities. It was observed (data not shown) that the structure prediction accuracy of TurboFold decreases when posterior alignment probabilities are utilized for generating extrinsic information instead of posterior *co-incidence *probabilities. In addition to combining information from homologous sequences, the extrinsic information can be generated experimentally. For example, in [58], the ability to use chemical mapping data is integrated into single sequence free energy minimization where it contributes to the structure prediction as an experimentally derived extrinsic information and is utilized in a non-iterative manner.

**Figure 10:**
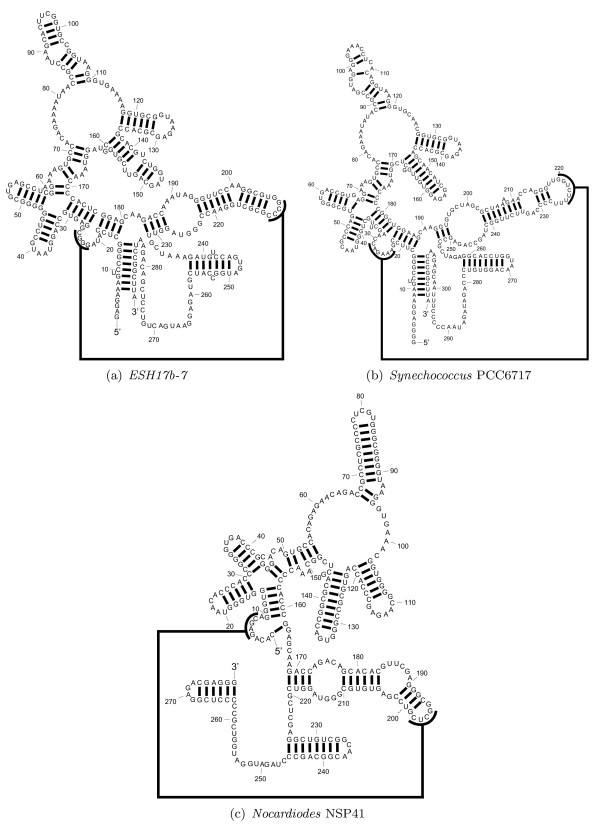
**Known structures for ESH17b-7, Synechococcus PCC6717, and Nocardiodes NSP41**. Known structures for *ESH17b-7, Synechococcus *PCC6717, and *Nocardiodes *NSP41 from the RNase P database [37]. 5' and 3' ends of sequences are indicated by "5'" and "3'". A heavy line between two nucleotides indicate the base pairing interaction between the nucleotides. A pseudoknot is indicated by a long thick line that connect the smaller thick lines, which are drawn along the paired nucleotides in the pseudoknot.

A major difference between TurboFold and most available programs is that TurboFold does not rigidly enforce commonality of secondary structure for the predictions across the multiple input sequences. This flexibility of TurboFold is in sharp contrast with algorithms in the Sankoff framework [32], where typically commonality of topology is rigidly enforced during the joint structure prediction process. The lack of a pre-defined model for commonality of secondary structures also distinguishes the method from alternative methods such as RNAcast [50,59] and RNA Sampler [49] that use representations of secondary structure to explore topologically equivalent foldings of multiple RNA sequences. When predicting structures for homologous sequences that have diverged significantly from each other, the ability of TurboFold to allow variable structure elements in some sequences offers an advantage. Variable structure elements are common in conserved RNA secondary structures [60]. One such example is the variable loop in tRNA, which can make a fifth arm in what is often a four-arm structure. An example of such a case in RNase P is shown in Figure 10, with the known secondary structures for three RNase P sequences, *ESH17b-7 *(GenBank accession number U28126), *Synechococcus *PCC6717 (X97392), and *Nocardiodes *NSP41 (AF110042[37]). The known structure for *Nocardiodes *NSP41 in Figure 10(c) diverged from the other structures with a four-way external loop between 5' and 3' ends. On the other hand, structures for *ESH17b-7 *and *Synechococcus *PCC6717 contain an external loop that contains a single branch with 5' and 3' dangling ends. Thus, the secondary structure for *Nocardiodes *NSP41 is topologically different, in terms of branching configurations, from the secondary structures for *ESH17b-7 *and *Synechococcus *PCC6717.

**Figure 11:**
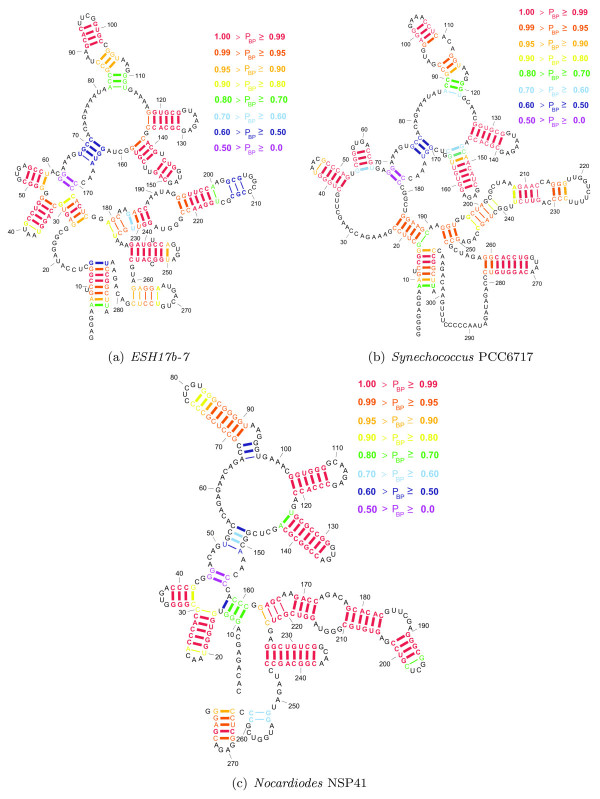
**TurboFold structure predictions for ESH17b-7, Synechococcus PCC6717, and Nocardiodes NSP41**. Structures for *ESH17b-7, Synechococcus *PCC6717, and *Nocardiodes *NSP41 as predicted by TurboFold. The heavy lines between nucleotides represent the correctly predicted base pairs and thin lines between nucleotides represents incorrectly predicted base pairs. The colors of thick lines indicate the probability of base pairing for the nucleotides as computed by TurboFold.

Figure 11 shows the structures for *ESH17b-7*, *Synechococcus *PCC6717, and *Nocardiodes *NSP41 as predicted by TurboFold. The external loop with multiple branches in the structure of *Nocardiodes *NSP41 is correctly predicted. Furthermore, the external loops in structures of *ESH17b-7*, *Synechococcus *PCC6717 are also correctly predicted.

**Figure 12:**
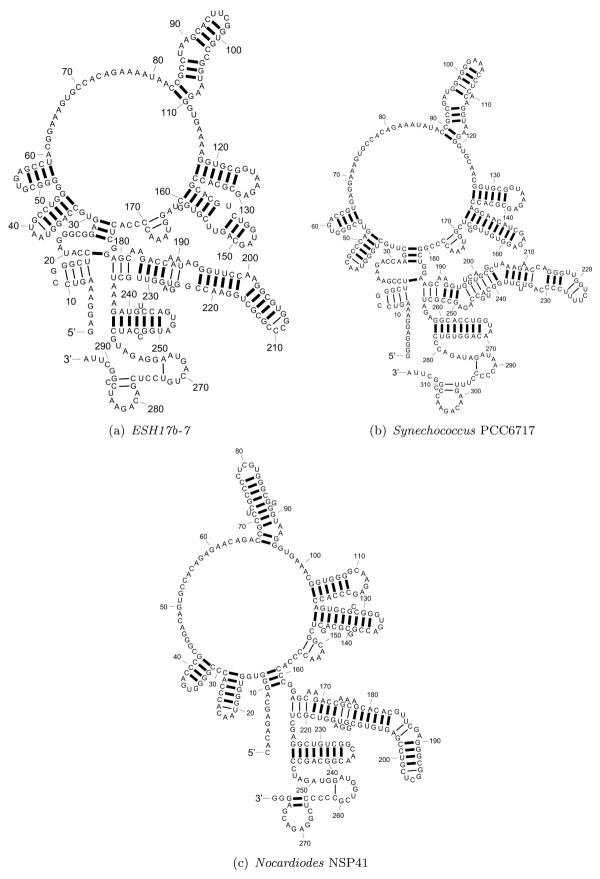
**RAF structure predictions for ESH17b-7, Synechococcus PCC6717, and Nocardiodes NSP41**. Structures for *ESH17b-7, Synechococcus *PCC6717, and *Nocardiodes *NSP41 as predicted by RAF. The heavy lines between nucleotides represent the correctly predicted base pairs and thin lines between nucleotides represents incorrectly predicted base pairs.

Figure 12 shows the structures for *ESH17b-7*, *Synechococcus *PCC6717, and *Nocardiodes *NSP41 as predicted by RAF. Although most of the predicted base pairs are consistent with the base pairs in known structures, the predicted structures are substantially different from the known structures in terms of the branching configurations of the structures: The external loop with multiple branches is predicted in all the structures. In addition, RAF predicts a 7 way multibranch loop between nucleotides at 29 - 174, 34 - 181, and 12 - 159 in structures of *ESH17b-7, Synechococcus *PCC6717, and *Nocardiodes *NSP41, respectively. The known structures comprise a helical domain at those nucleotides positions, which are followed by two 4 way multibranch loops. Note that the topologies of these domains are correctly predicted by TurboFold. TurboFold predicts individual RNA secondary structures using extrinsic information from homologous sequences. This problem is closely related to but not identical to the problem of predicting consensus structures for the homologs. The benchmarks in Figures 8 and 9 are scored on the known secondary structures of individual RNAs rather than on the consensus structures and therefore somewhat unfair to consensus structure methods included in the tables; ideally consensus structure methods should be scored only on consensus structures. The benchmarking methodology is adopted in order to facilitate comparison of the methods despite the fact they address different problems. An alternative method would be to convert the consensus predictions into individual RNA structure predictions by folding the RNAs while using the consensus structure as a constraint. This allows non-conserved pairs to be added as long as they are consistent with the consensus and improves sensitivity at the cost of PPV. The method, however, introduces additional dependence on how exactly the constrained folding is performed and is therefore not considered here.

The inverse similarity weighting (1 - *ψ*_*m*,*s*_) in (2) is a good choice despite the fact that fact that, under this weighting, larger weights are assigned to highly diverged sequences can often not be aligned well. This is because, unlike methods that determine one alignment and incorporate it in jointly folding sequences, the alignment information in TurboFold is probabilistic and incorporated in the form of nucleotide co-incidence probabilities. For highly diverged sequences, these co-incidence probabilities are smaller in magnitude and diffused over a wider region. Though the inverse similarity weighting (1 - *ψ*_*m*,*s*_) in (2) assigns larger weights to highly diverged sequences, they do not exercise a strong influence when the extrinsic information is computed by averaging across multiple sequences in (2). The experimental results for SRP sequences, whose predicted average pairwise identity is 42%, are in agreement with this observation. Compared with other methods TurboFold predictions provide the highest sensitivity.

The concept of iterative updates utilized in TurboFold is motivated by iterative error-correction coding methods in digital communications [61], especially *Turbo decoding *[28,62]. For the case of two RNA homologs, based on the analogy with turbo decoding, the conceptual framework for iterative estimation of RNA secondary structures and alignments was previously introduced by the authors in [63], albeit without a practical realization and also with significant differences in details. Both TurboFold and Turbo decoding rely on multiple encodings of common underlying information, which the estimation (decoding) procedures seek to recover. In TurboFold a (largely) common secondary structure is "encoded" by nature in the form of multiple homologous sequences and the goal of the estimation is to recover this common secondary structure. In Turbo decoding, a common digital data stream is deliberately encoded by multiple, usually two, encoders prior to communication over a channel and the receiver seeks to recover the common digital data stream. Both problems benefit from iterative update procedures that are enabled by re-framing decoding or prediction in terms of estimating corresponding probabilities. Specifically, in TurboFold, the formulation of the RNA folding problem as a base pairing probability estimation problem, as opposed to the problem of estimating one or more consensus secondary structures, allows propagation of probabilistic information from one sequence to the other and iterative updates. It is also noteworthy that in TurboFold the extrinsic information is incorporated as a free energy modification in the partition function for estimating single sequence base pairing probabilities with minimal computational cost, which is analogous, in Turbo decoding, to the method for insertion of extrinsic information as a pseudo prior [62] in the decoding procedure for the recovery of a single encoded stream. There are also obvious differences between TurboFold and Turbo decoding. Whereas, in Turbo decoding, the encoding of the data is designed manually for explicitly enabling recovery at the receiver, there is no such explicit design for the multiple homologs that form the input to TurboFold. This apparent disadvantage is, however, offset in part by the fact that typically many more homologs are available for an ncRNA sequence for use in TurboFold whereas in Turbo decoding use of more than two encodings levies a cost in power and data rate that is usually not justified by relatively minor performance gains that these additional encodings enable.

The main limitation of TurboFold is its inability to predict sequence alignments that conform to the predicted secondary structures. In parallel with previously proposed iterative decoding of RNA structural alignment in [64], the most natural extension of TurboFold for prediction of sequence alignment is via an integration of a probabilistic model for alignment into the existing iterative structure prediction. A probabilistic model for alignment already exists in the hidden Markov model. The iterations, however, do not update the co-incidence probabilities of alignment. The integration of probabilistic alignment model into the iterative prediction is currently a future consideration.

## Conclusion

TurboFold, a new method for secondary structure prediction for multiple homologous sequences, is presented in this paper. TurboFold estimates base pairing probabilities for each of the sequences via an iterative procedure that utilizes extrinsic information from other sequences and intrinsic information from a thermodynamic nearest neighbor model for RNA folding. Experimental results demonstrate that the iterative updates in TurboFold offer a significant improvement over both single sequence computations and over non-iterative multi-sequence computations of base pairing probabilities. The base pairing probability estimates from TurboFold outperform alternative multi-sequence methods for estimating base pairing probabilities. TurboFold can be downloaded, either as source code or precompiled binaries as part of the RNAstructure package for Microsoft Windows, at http://rna.urmc.rochester.edu.

## Authors' contributions

AOH designed the studies, wrote the TurboFold code, performed the experiments, and drafted the manuscript. GS and DHM conceived the studies and revised the manuscript. All authors read and approved the final manuscript.

## TurboFold Algorithm

**input **: A set of *K *homologous RNA sequence ,  = {1,2, ..., *K*}.

**output**: Posterior base pairing probability estimates  for each RNA sequence in the set.

begin

   **for ***m *← 1 **to ***K ***do**

      **for ***s *← 1 **to ***K ***do**

         // Compute the alignment co-incidence probabilities and sequence identities via a hidden Markov pairwise sequence alignment model

         Compute *_c_***Π **^(*s*,*m*) ^and *ψ*_*s,m*_;

      **end**

   **end**

   // Iterate (*η *+1) times.

   t ← 0;

   **while **t ≤ *η ***do**

      **for ***m *← 1 **to ***K ***do**

         // Compute extrinsic information for base pairing

         **if **(*t *== 0) **then**

            // Use uniform unity initialization for extrinsic information

            ;

         **else**

            Compute  utilizing  (details in Figures 2, 3, 4);

         **end**

         // Compute base pairing probabilities via modified partition function computation

         Compute  utilizing  and nearest neighbor thermodynamic model;

      **end**

      *t *← *t *+ 1;

   **end**

end

**Algorithm 1**: TurboFold: Iterative probabilistic structure prediction

## Supplementary Material

Additional file 1**Supplementary Material for "TurboFold: Iterative Probabilistic Estimation of Secondary Structures for Multiple RNA Sequences,"**. This file contains supplementary information for the manuscript that provides details for the computation of the normalization factor, , in Eqn. (2), plots of sensitivity versus PPV for predictions of TurboFold over tRNA and 5S rRNA training datasets with *K *= 10 for varying values of *η*, number of iterations, and *γ*, weight of extrinsic information, parameters, and plots of number sequences (*K*) versus sensitivity and versus PPV for testing datasets stratified in terms of sequence identity.Click here for file

Additional file 2 **Zip file with TurboFold source code**. Version of the TurboFold source code at time of publication of this paper. Click here for file

Additional file 3 **Zip file of datasets**. Datasets used in the benchmarking of TurboFold and other algorithms. Click here for file
