# Supplementary material for: TurboFold: Iterative probabilistic estimation of secondary structures for multiple RNA sequences
Source: BMC Bioinformatics. 2011 Apr 20;12:108. doi: 10.1186/1471-2105-12-108 (PMC3120699; doi:10.1186/1471-2105-12-108)
Supplement: Additional file 1 — Supplementary Material for "TurboFold: Iterative Probabilistic Estimation of Secondary Structures for Multiple RNA Sequences,". This file contains supplementary information for the manuscript that provides details for the computation of the normalization factor, , in Eqn. (2), plots of sensitivity versus PPV for predictions of TurboFold over tRNA and 5S rRNA training datasets with K = 10 for varying values of η, number of iterations, and γ, weight of extrinsic information, parameters, and plots of number sequences (K) versus sensitivity and versus PPV for testing datasets stratified in terms of sequence identity. [file 1471-2105-12-108-S1.PDF]

# Supplementary Material for "TurboFold: Iterative Probabilistic Estimation of Secondary Structures for Multiple RNA Sequences," published in *BMC Bioinformatics*, vol 12, 2011.

Arif Ozgun Harmanci[1,4], Gaurav Sharma[1,3,4,*], David H. Mathews[2,3,4]

[1] *Department of Electrical and Computer Engineering, University of Rochester, Rochester, NY, USA*

[2] *Department of Biochemistry and Biophysics, University of Rochester Medical Center, Rochester, NY, USA*

[3] *Department of Biostatistics and Computational Biology, University of Rochester Medical Center, Rochester, NY, USA*

[4] *Center for RNA Biology, University of Rochester, Rochester, NY, USA*

## Overview

The supplementary material includes the computation of the normalization factor, ${}_p^t\alpha^m$, in Equation (2) in the main text, plots of sensitivity versus PPV for predictions of TurboFold over tRNA and 5S rRNA training datasets with $K = 10$ for varying values of $\eta$, number of rounds, and $\gamma$, weight of extrinsic information, parameters, and plots of number sequences ($K$) versus sensitivity and versus PPV for testing datasets stratified in terms of sequence identity.

## Computation of ${}_p^t\alpha^m$ in Equation (2)

Equation (2) in the main text is included below for the reader's convenience:

$$ {}_p^t\tilde{\mathbf{\Pi}}^m = {}_p^t\alpha^m \sum_{s\in\mathcal{N}\setminus m} (1-\psi_{m,s}) \; {}_p^{t-1}\tilde{\mathbf{\Pi}}^{(s\to m)} \tag{1} $$

where ${}_p^t\alpha^m$ is the normalizing factor to set the maximum of ${}_p^t\tilde{\mathbf{\Pi}}^m$ to 1.

To obtain ${}_p^t\alpha^m$, first an unnormalized version of the matrix on the right hand side of (1) is computed, i.e.

$$ {}_p^t\bar{\mathbf{\Pi}}^m = \sum_{s\in\mathcal{N}\setminus m} (1-\psi_{m,s}) \; {}_p^{t-1}\tilde{\mathbf{\Pi}}^{(s\to m)} \tag{2} $$

Then ${}_p^t\alpha^m$ is obtained as the maximum value over this unnormalized matrix, i.e.

$$ {}_p^t\alpha^m = \max_{i,j} \left[{}_p^t\bar{\mathbf{\Pi}}^m\right]_{ij} \tag{3} $$

## Sensitivity versus PPV Plots for Predictions over $K = 10$ Datasets

Sensitivity versus PPV plots for predictions of TurboFold with varying $\eta$ and $\gamma$ parameters over training datasets with $K = 10$ are shown in Fig. S.13 and in Fig. S.14, respectively.

*Corresponding Author.
*Email addresses:* arharman@ece.rochester.edu (Arif Ozgun Harmanci), gaurav.sharma@rochester.edu (Gaurav Sharma), David_Mathews@urmc.rochester.edu (David H. Mathews).

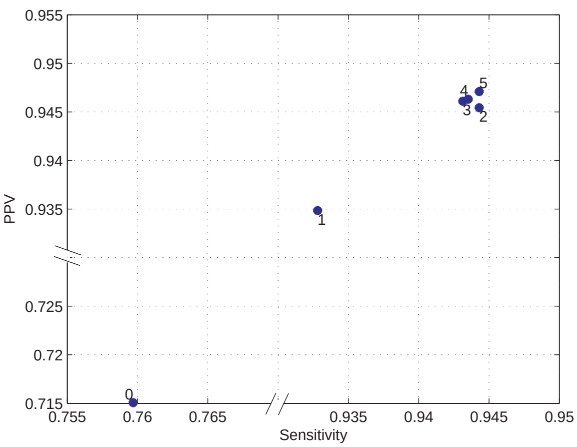


(a) tRNA

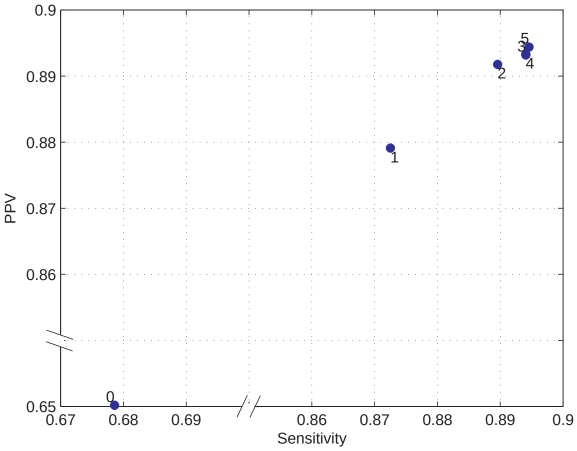


(b) 5S rRNA

Figure S.13: Plots of sensitivity versus PPV for structure prediction by TurboFold with increasing value of $\eta$, number of iterations, for: (a) tRNA and (b) 5S rRNA training datasets with $K = 10$. Note the discontinuities in the axes which are indicated by the breaks.

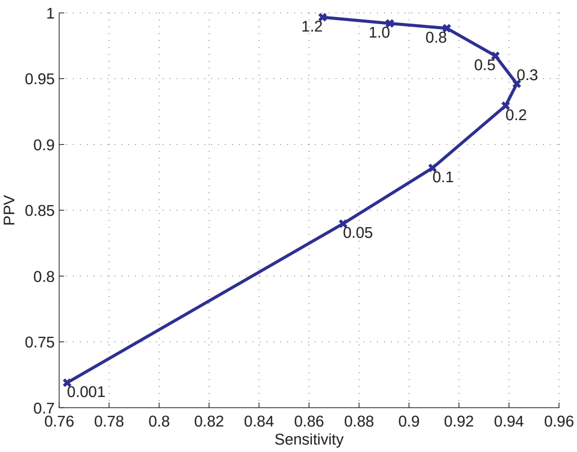


(a) tRNA

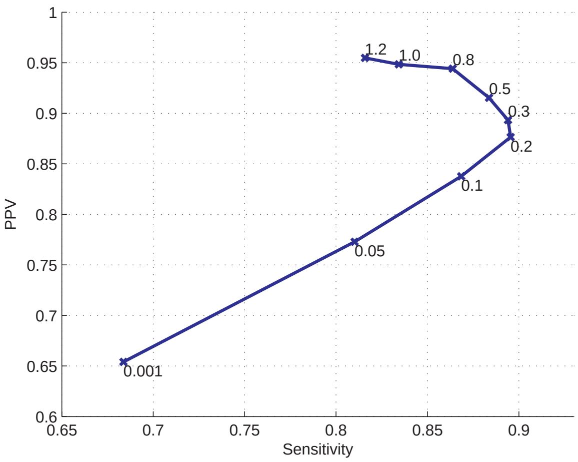


(b) 5S rRNA

Figure S.14: Plots of sensitivity versus PPV for structure prediction by TurboFold with increasing value of $\gamma/RT$ for: (a) tRNA and (b) 5S rRNA training datasets with $K = 10$.

## Sensitivity versus PPV Variation with Sequence Identity

The datasets are stratified in terms of the average sequence identity as estimated from the maximum likelihood alignments computed by the pairwise alignment hidden Markov model with steps of 0.5. $K$ versus sensitivity and $K$ versus PPV plots in Figuress 7 and 8 in the main text are plotted for the stratified datasets in Figs. S.15 and S.16.

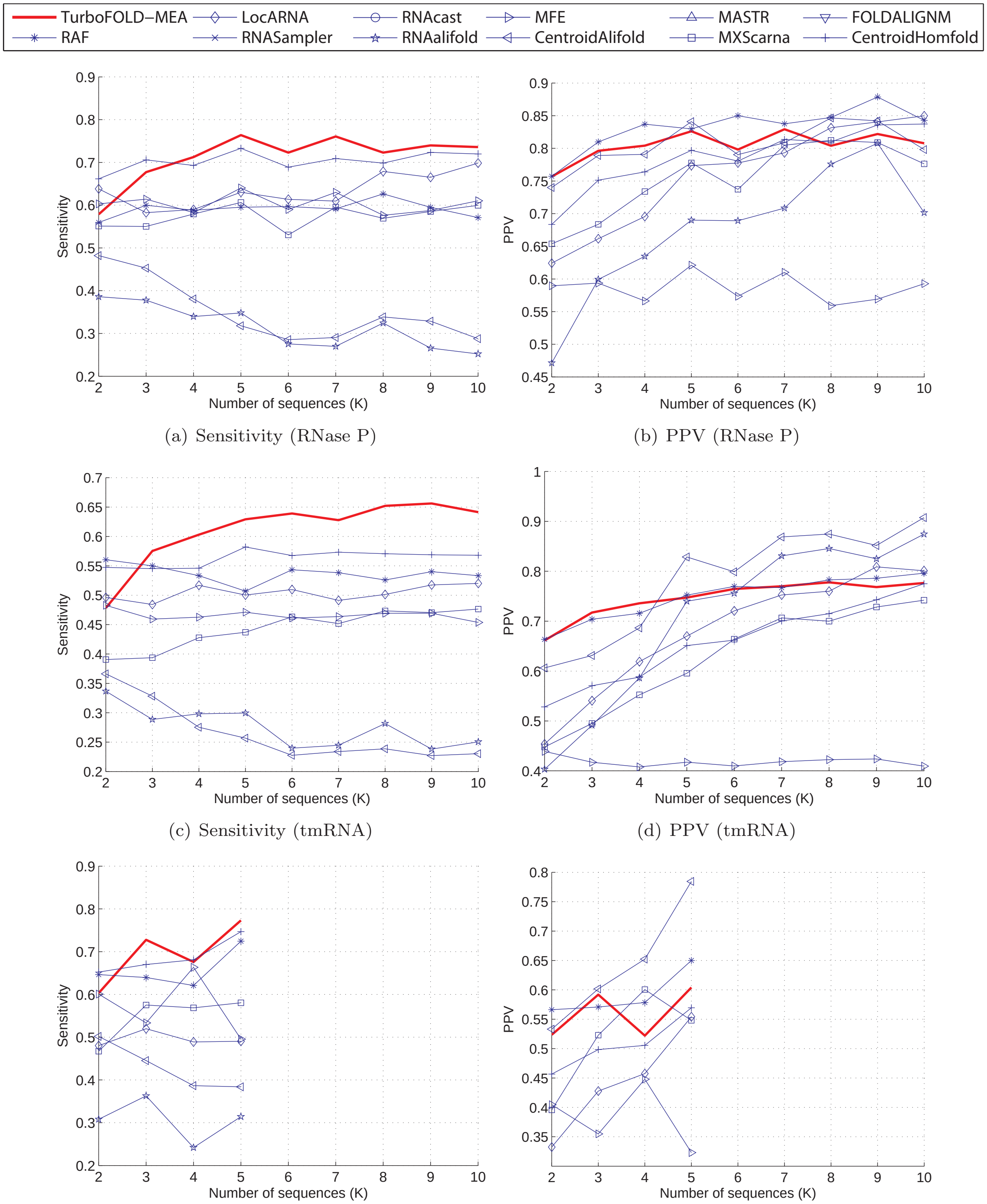


(a) Sensitivity (RNase P)

(b) PPV (RNase P)

(c) Sensitivity (tmRNA)

(d) PPV (tmRNA)

(e) Sensitivity (telomerase RNA)

(f) PPV (telomerase RNA)

Figure S.15: Accuracy of structure prediction versus number of sequences for datasets with average pairwise identity between 0 and 0.5. Sensitivity and PPV for RNase P, (a) and (b), respectively, tmRNA, (c) and (d), respectively, and telomerase RNA, (e) and (f), respectively, datasets with average pairwise identity between 0 and 0.5. Methods that did not complete execution for a dataset because memory requirements exceeded available resources are excluded from the corresponding plots. For Telomerase datasets, for $K$ greater than 5, the datasets did not include any $K$ sequence sets for which the average estimated identity lower than 0.5.

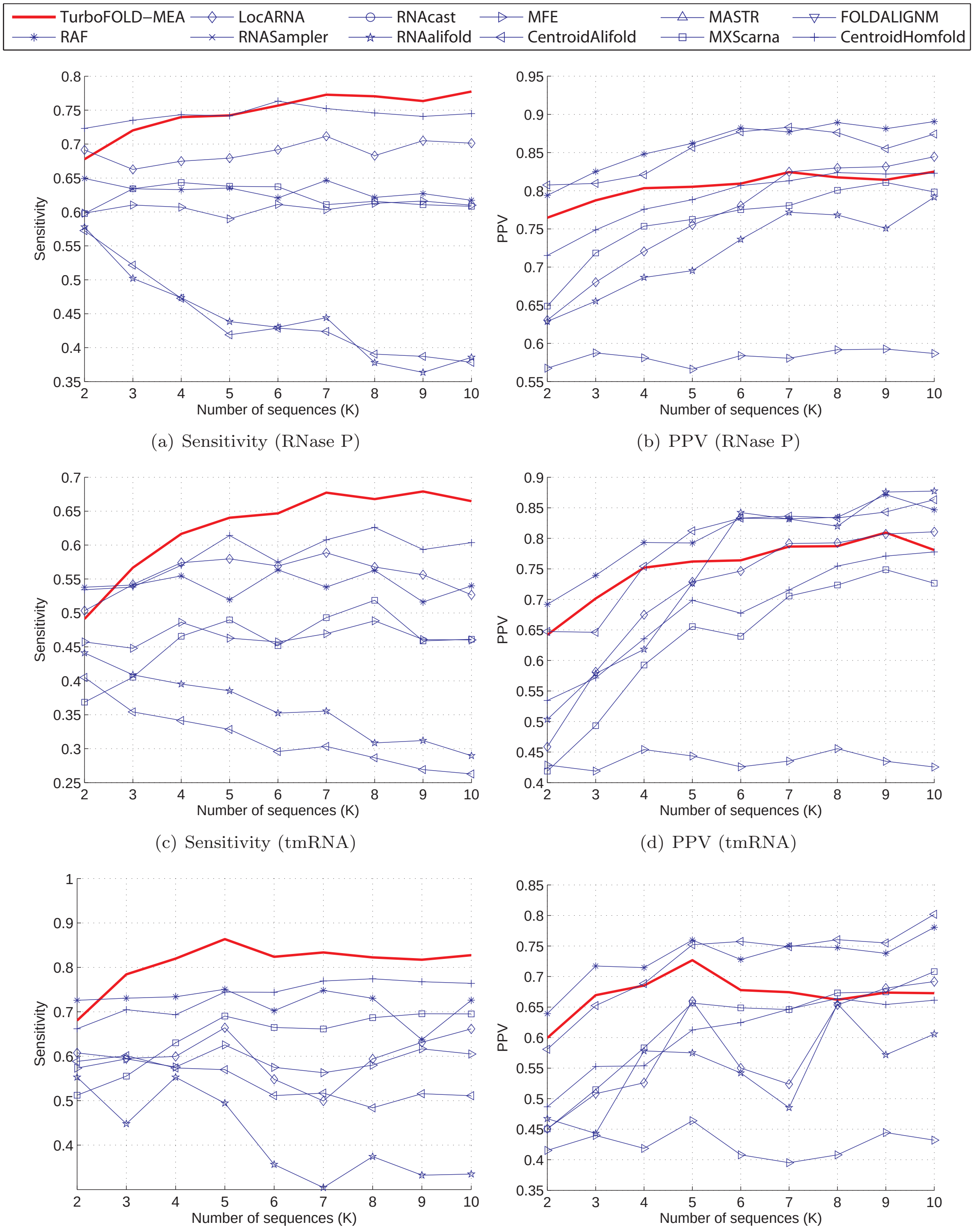


(a) Sensitivity (RNase P)

(b) PPV (RNase P)

(c) Sensitivity (tmRNA)

(d) PPV (tmRNA)

(e) Sensitivity (telomerase RNA)

(f) PPV (telomerase RNA)

Figure S.16: Accuracy of structure prediction versus number of sequences for datasets with average pairwise identity between 0.5 and 1. Sensitivity and PPV for RNase P, (a) and (b), respectively, tmRNA, (c) and (d), respectively, and telomerase RNA (e) and (f), respectively. Methods that did not complete execution for a dataset because memory requirements exceeded available resources are excluded from the corresponding plots.

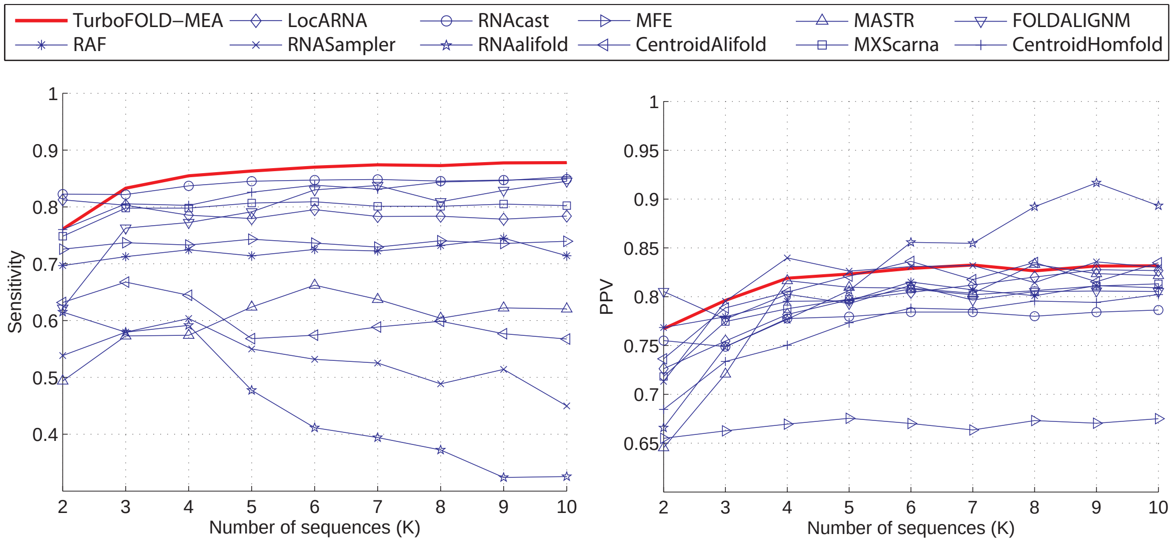


(a) Sensitivity (SRP)

(b) PPV (SRP)

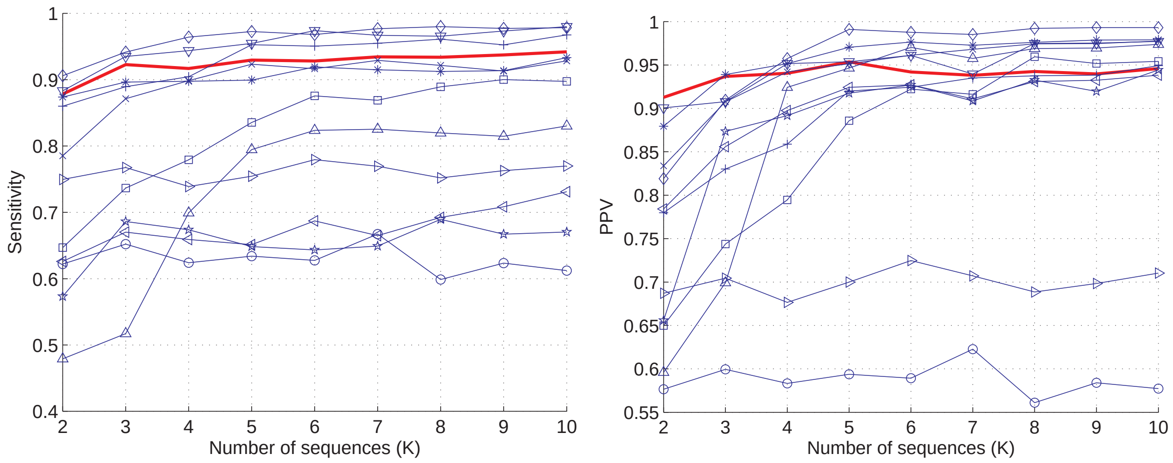


(c) Sensitivity (tRNA)

(d) PPV (tRNA)

Figure S.17: Sensitivity and PPV of structure prediction versus number of sequences for SRP ((a) and (b), respectively) and tRNA ((b) and (c), respectively) datasets with average pairwise identity between 0 and 0.5. Methods that failed prediction for any dataset are excluded from the corresponding accuracy plots. Also, 5S rRNA plots are not included because for all the $K$ sequences in the dataset the estimated average pairwise identity is greater than 0.50.

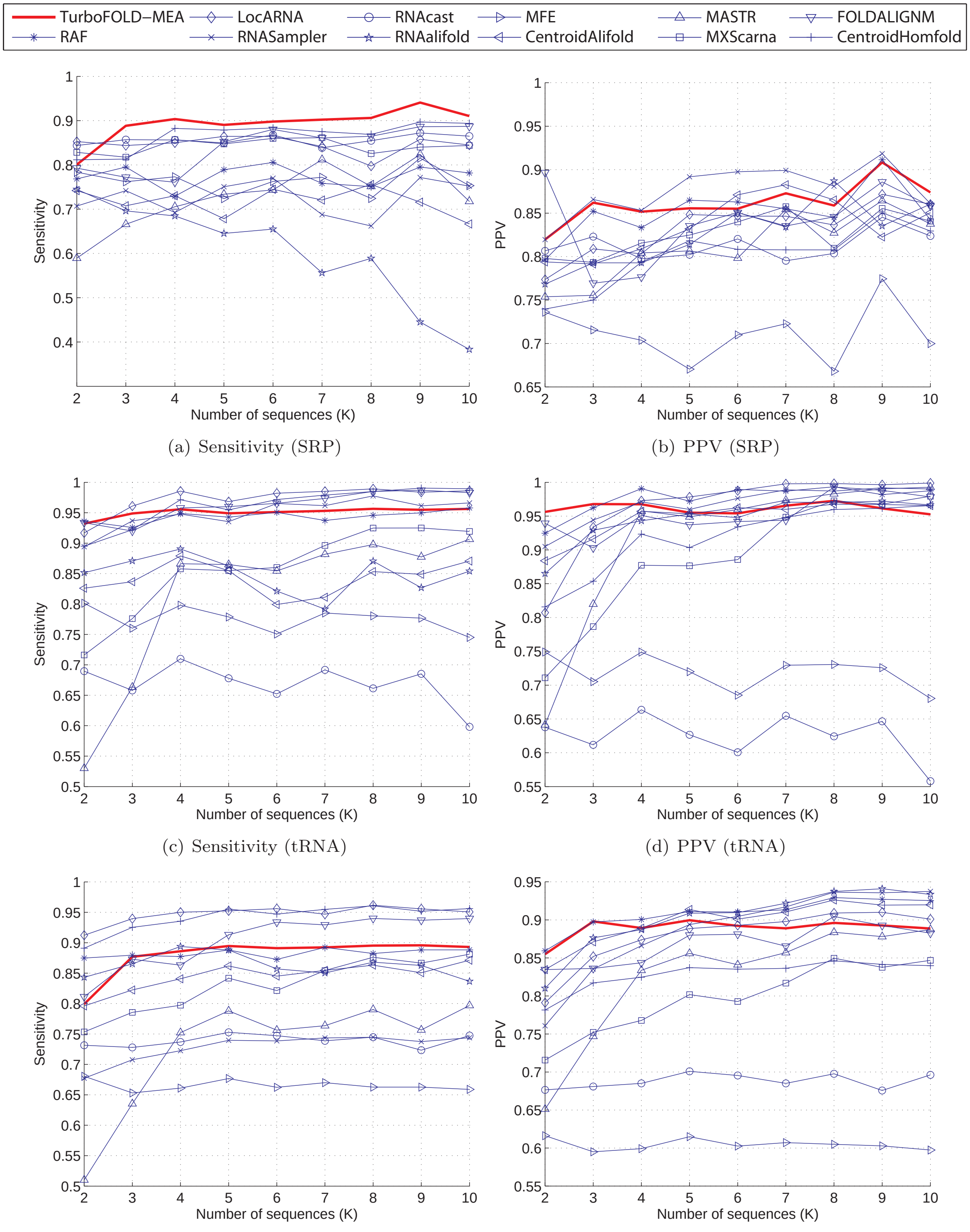


(a) Sensitivity (SRP)

(b) PPV (SRP)

(c) Sensitivity (tRNA)

(d) PPV (tRNA)

(e) Sensitivity (5S rRNA)

(f) PPV (5S rRNA)

Figure S.18: Sensitivity and PPV of structure prediction versus number of sequences for SRP ((a) and (b), respectively), tRNA ((b) and (c), respectively), and 5S rRNA ((d) and (e), respectively) datasets with average pairwise identity between 0.50 and 1. Methods that failed prediction for any dataset are excluded from the corresponding accuracy plots.
